# Supplementary material for: Rescue of astrocyte activity by the calcium sensor STIM1 restores long-term synaptic plasticity in female mice modelling Alzheimer’s disease
Source: Nat Commun. 2023 Mar 22;14:1590. doi: 10.1038/s41467-023-37240-2 (PMC10033875; doi:10.1038/s41467-023-37240-2)
Supplement: Supplementary file 1 — Supplementary Information [file 41467_2023_37240_MOESM1_ESM.pdf]

Rescue of astrocyte activity by the calcium sensor STIM1 restores long-term synaptic plasticity in female Alzheimer's disease mice (Lia et al.)

Supplementary Information

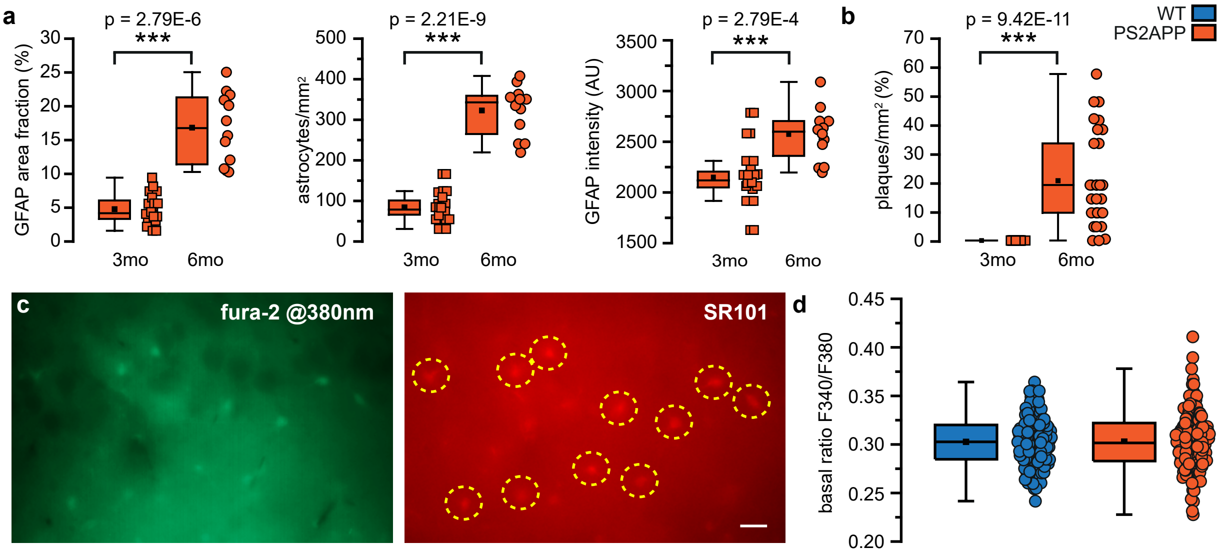

Supplementary Fig. 1. Quantification of gliosis and plaque deposition in 3mo and 6mo PS2APP mice and basal $[Ca^{2+}]_{cyt}$ level in 6mo WT and PS2APP mice. (**a**) Left, area fraction covered by GFAP immunostaining signal. Middle, number of GFAP-positive astrocytes per mm^2. Right, mean gray value of GFAP intensity. Measurements obtained from maximum Z-projections (Z-depth 8 μm) of confocal images from layers I-VI of the SSCx as in Fig. 1b. Box plots show mean, median, 25th and 75th percentile, outliers. n = 16 fields from 2 PS2APP mice (3mo); n = 12 fields from 2 PS2APP mice (6mo). (**b**) Number of 4G8-positive plaques/mm^2 counted in maximum Z-projections (Z-depth 8 μm) of confocal images from layers I-VI of the SSCx. Box plots show mean, median, 25th and 75th percentile, outliers. n = 29 fields from 3 PS2APP mice (3mo); n = 30 fields from 8 PS2APP mice (6mo). (**c**) Left, basal fura-2 signal at 380nm in representative SSCx astrocytes from a 6mo PS2APP mouse. The image is an average projection of 60 frames from a t-series. Right, SR101 fluorescence in the same field. Yellow circles identify fura-2/SR101 positive astrocytes. Scalebar 20 μm. (**d**) Box and scatter plots of the ratio of fura-2 emitted fluorescence (F340/F380) in individual astrocytes in resting conditions from 6mo WT and PS2APP mice. Box plots show mean, median, 25th and 75th percentile, outliers. n = 203 astrocytes from 3 WT mice and n = 168 astrocytes from 3 PS2APP mice. *** $p < 0.001$, Two-tailed two sample Student's t-test (a), two-tailed Mann-Whitney test (b, d). Source data are provided as a Source Data file.

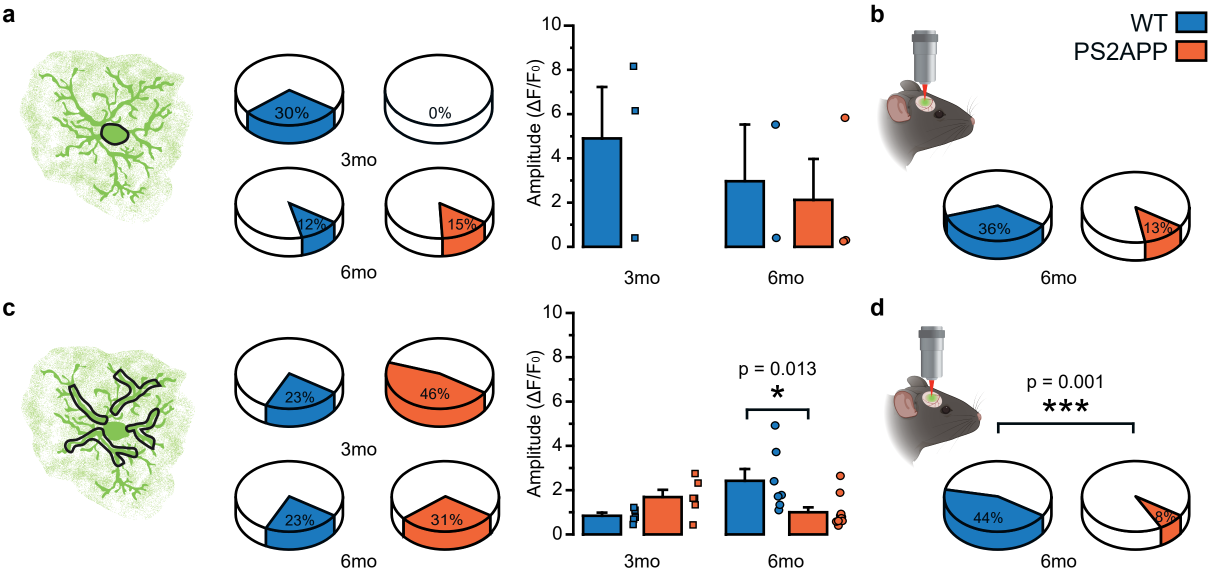

Supplementary Fig. 2. Spontaneous astrocyte Ca^{2+} activity in SSCx brain slices and *in vivo* at soma and proximal processes. (**a**) Left, pie charts reporting the percentage of active somata; right, bar histograms and scatter plots (mean ± SEM) reporting response amplitude in SSCx brain slices. 3mo: n = 10 astrocytes from 4 WT and 8 astrocytes from 4 PS2APP mice; 6mo: n = 17 astrocytes from 6 WT and 20 astrocytes from 7 PS2APP mice. For amplitude only responsive somata have been considered. (**b**) Pie charts reporting the percentage of active somata in *in vivo* preparations; n = 11 astrocytes from 3 WT and 22 astrocytes from 3 PS2APP 6-month-old mice. (**c**) Left, pie charts reporting the percentage of active proximal processes; right, bar histograms (mean ± SEM) and scatter plots reporting response amplitude in SSCx brain slices. 3mo: n = 8 astrocytes from 4 WT and 8 astrocytes from 4 PS2APP mice; 6mo: n = 14 astrocytes from 6 WT and 17 astrocytes from 6 PS2APP mice. For amplitude only astrocytes with at least one responsive proximal process have been considered. (**d**) Pie charts reporting the percentage of active proximal processes in *in vivo* preparations; n = 11 astrocytes from 3 WT and 13 astrocytes from 3 PS2APP 6mo mice. *$p < 0.05$, ***$p < 0.001$, Two-tailed Mann-Whitney test for bar histograms and Fisher's exact test for pie-charts. Source data are provided as a Source Data file.

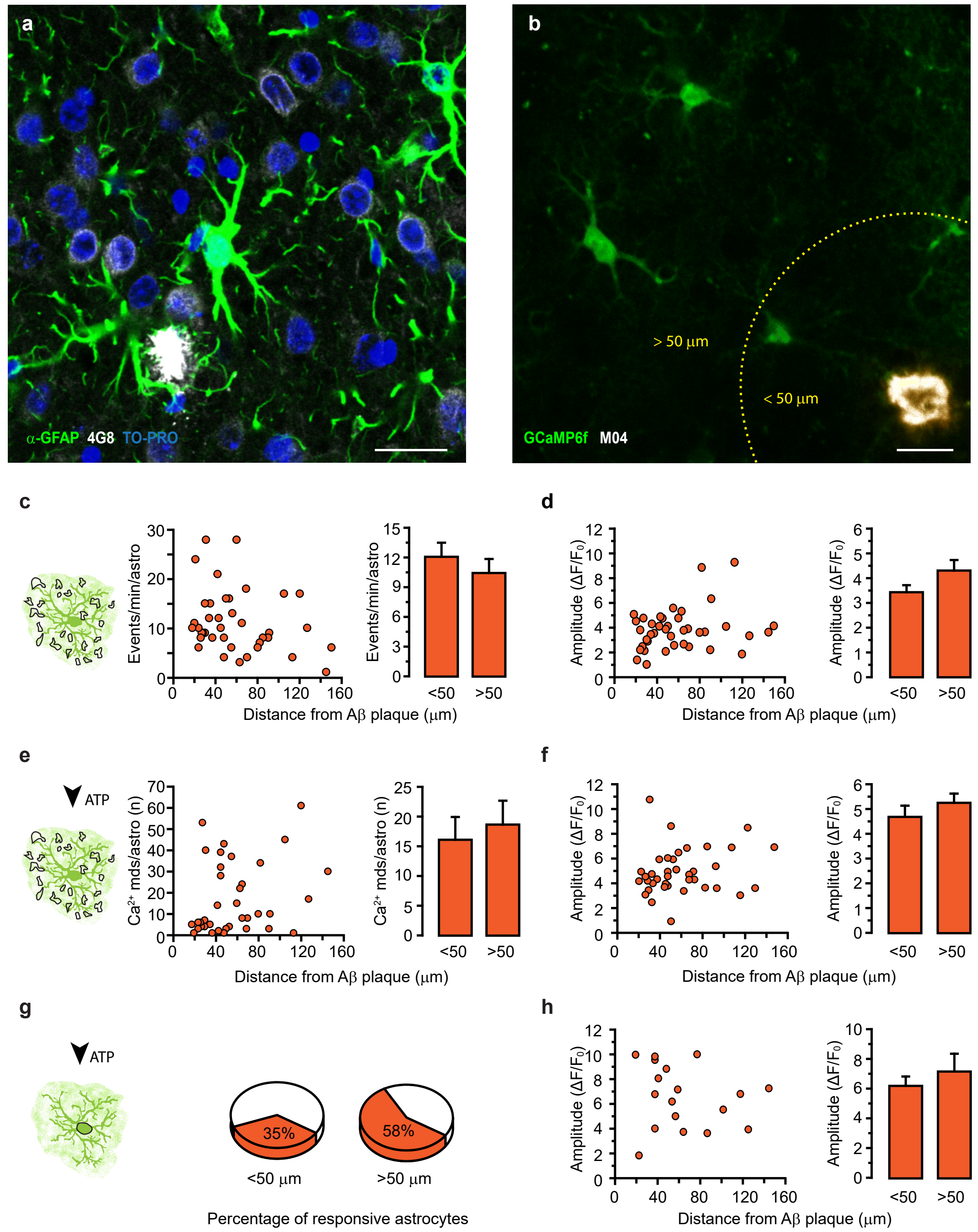

Supplementary Fig. 3. Relationship between astrocyte Ca^{2+} hypoactivity and Aβ-plaque proximity in 6mo PS2APP mice. (**a**) Confocal image of SSCx stained with α-GFAP (green) for astrocytes, 4G8 (white) for Aβ-plaques and TO-PRO-3 (blue) for nuclei (representative of 3 mice). Scale bar 20 μm. (**b**) 2P image of SSCx showing GCaMP6f-expressing astrocytes and Aβ-plaque marked by M04 (bottom right corner, white; representative of 6 mice). The dashed yellow line divides astrocytes within or above 50 μm from the Aβ-plaque. Scale bar 20 μm. (**c**) Scatter matrix of spontaneous md frequency plotted against distance from Aβ-plaque (Spearman's correlation (SC) r_s = 0.25). Right, bar histograms of md frequency (mean ± SEM) depending on Aβ-plaque distance. n = 40 astrocytes, 19 within 50 μm and 21 above 50 μm from Aβ-plaque, 6 mice. (**d**) Same as (c) but for spontaneous md amplitude (SC r_s = 0.16). (**e**) Scatter matrix of ATP-evoked mds plotted against Aβ-plaque distance (SC r_s = -0.21). Right, bar histograms of md number (mean ± SEM) depending on Aβ-plaque distance. n = 38 astrocytes, 20 within 50 μm and 18 above 50 μm from Aβ-plaque, 6 mice. (**f**) Same of (e) but for ATP-evoked md amplitude (SC r_s = 0.20). Right, bar histograms of md amplitude (mean ± SEM) depending on Aβ-plaque distance. (**g**) Pie charts reporting the percentage of responding somata with respect to Aβ-plaque distance. n = 18 astrocytes, 7 within 50 μm and 11 above 50 μm from Aβ-plaque, 3 mice. (**h**) Same as (f) but for somata (SC r_s = 0.21). Two-tailed Mann-Whitney test for bar histograms and Fisher's Exact test for pie charts. Source data are provided as a Source Data file.

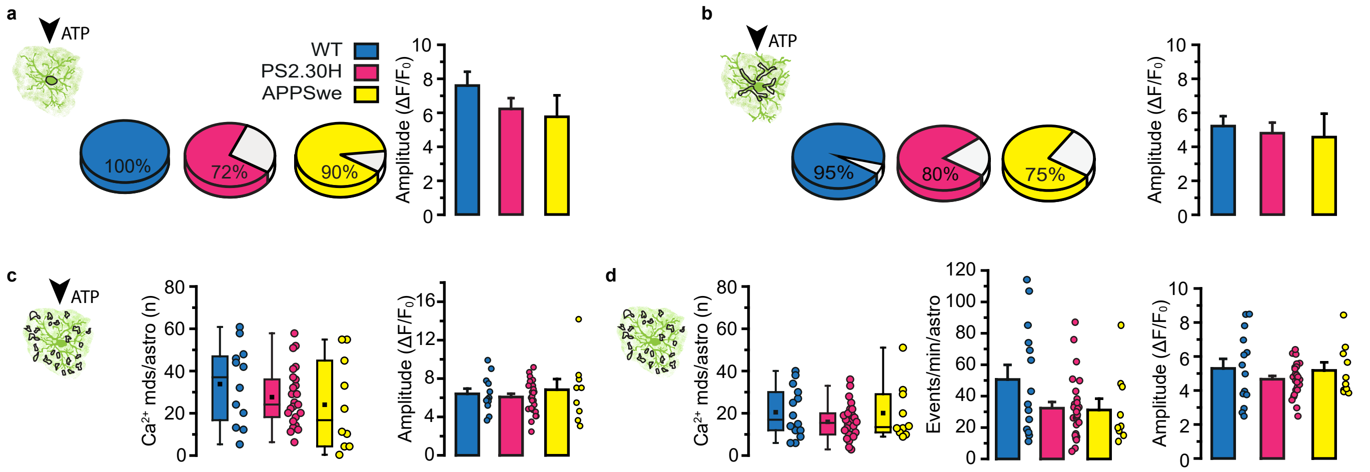

Supplementary Fig. 4. ATP-evoked and spontaneous astrocyte Ca^{2+} signals in SSCx from 6mo PS2.30H and APPSwe mice. (**a**) Pie charts reporting the percentage of ATP responsive somata and bar histograms reporting the response amplitude (mean ± SEM) in 6mo WT (blue), PS2.30H (magenta) and APPSwe (yellow) mice. n = 12 astrocytes from 6 WT, 22 astrocytes from 7 PS2.30H and 10 astrocytes from 3 APPSwe mice. For amplitude only responsive somata have been considered. (**b**) Same as (a) but for proximal processes. n = 12 astrocytes from 6 WT, 26 astrocytes from 7 PS2.30H and 10 astrocytes from 3 APPSwe mice. For amplitude only astrocytes with at least one responsive proximal process have been considered. (**c**) Box and scatter plots of ATP-evoked Ca^{2+} mds and bar histograms (mean ± SEM) and scatter plots reporting the response amplitude. Box plots report mean, median, 25th and 75th percentile, outliers. n= 12 astrocytes from 6 WT, 25 astrocytes from 7 PS2.30H and 10 astrocytes from 3 APPSwe mice. For amplitude only astrocytes with at least one Ca^{2+} md have been considered. (**d**) Box and scatter plots of spontaneous Ca^{2+} mds and bar histograms (mean ± SEM) and scatter plots reporting event frequency and amplitude. Box plots report mean, median, 25th and 75th percentile, outliers. n= 14 astrocytes from 6 WT, 26 astrocytes from 7 PS2.30H and 10 astrocytes from 4 APPSwe mice. For amplitude and number of events, only astrocytes with at least one active Ca^{2+} md have been considered. One-way ANOVA (a, c), Kruskal Wallis ANOVA (b, d). Source data are provided as Source Data file.

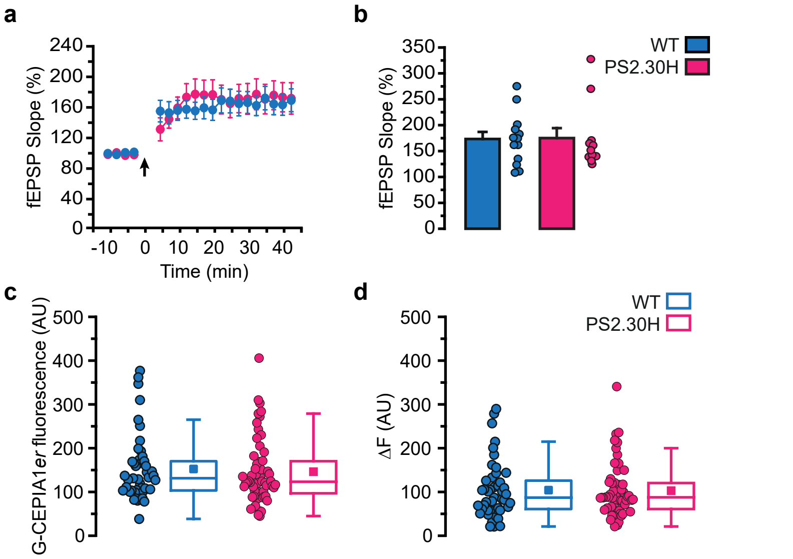

Supplementary Fig. 5. LTP and astrocyte $[Ca^{2+}]_{ER}$ levels in SSCx from 6mo PS2.30H mice. (**a**) fEPSP slope before and after TBS (black arrow) in SSCx slices from 6mo WT (blue) and PS2.30H (magenta) mice (mean ± SEM). n = 13 slices from 9 WT and 11 slices from 5 PS2.30H mice. (**b**) Bar histograms (mean ± SEM) and scatter plots of fEPSP slope. n = 13 slices from 9 WT and 11 slices from 5 PS2.30H mice. (**c**) Scatter and box plots of astrocyte somata G-CEPIA1*er* fluorescence values in SSCx slices from 6mo WT and PS2.30H mice. Box plots show mean, median, 25th and 75th percentile, outliers. n = 45 astrocytes from 8 WT and 56 astrocytes from 2 PS2.30H mice. (**d**) Scatter and box plots of astrocyte soma G-CEPIA1*er* fluorescence drop (ΔF) values upon CPA perfusion in SSCx slices. Box plots show mean, median, 25th and 75th percentile, outliers. n = 45 astrocytes from 8 WT and 56 astrocytes from 2 PS2.30H mice. Two-tailed two-sample Student's t-test (b) and two-tailed Mann-Whitney test (c, d). Source data are provided as a Source Data file.

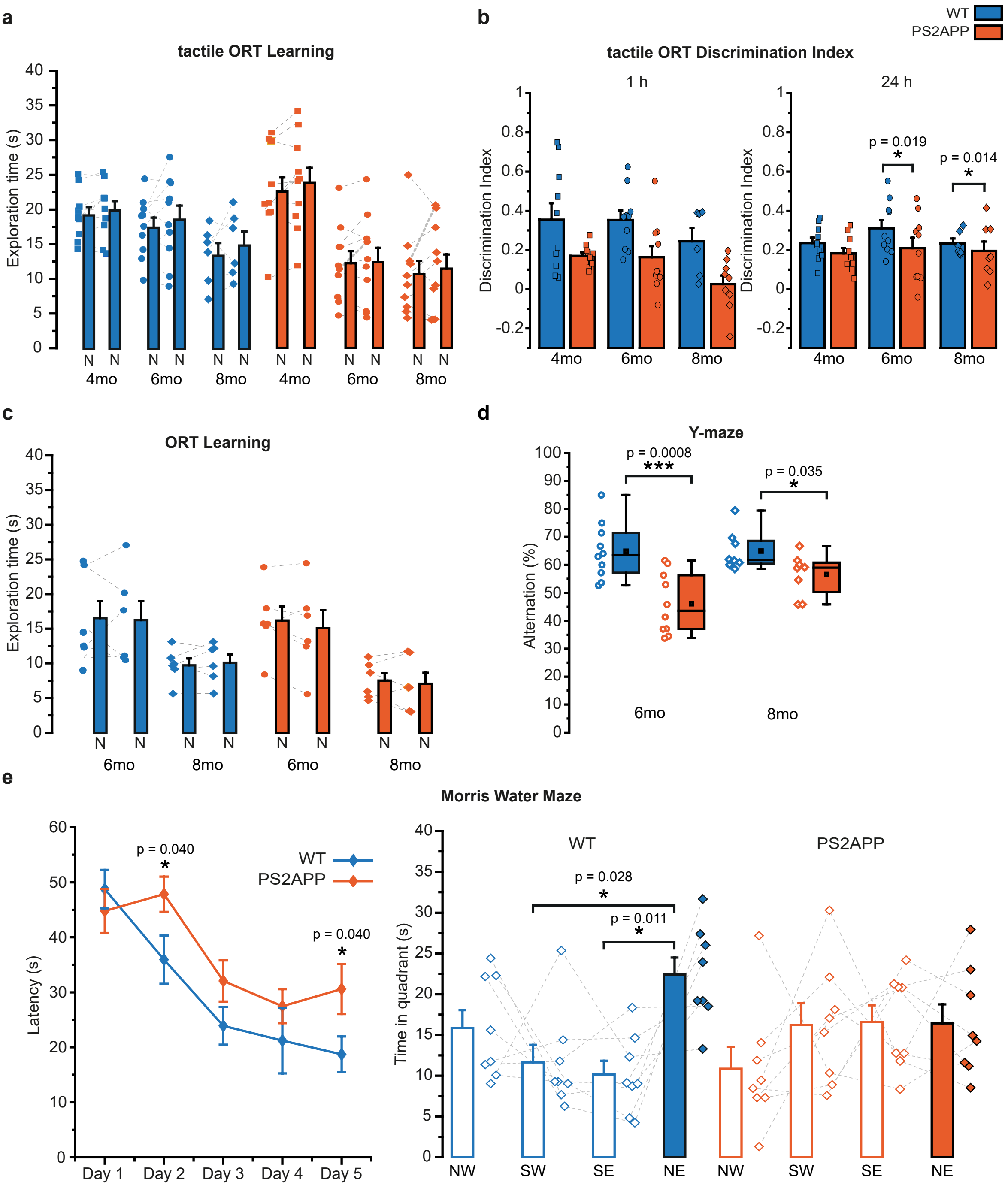

Supplementary Fig. 6. Behavioural analysis in WT and PS2APP mice. (**a**) Bar histograms reporting exploration time (mean ± SEM) of the two novel (N) objects during the learning phase of tORT in WT and PS2APP mice at 4, 6 and 8 months. Individual values are reported as symbols connected by a dashed line (4mo and 6mo: n = 10 WT and 10 PS2APP mice; 8mo: n = 6 WT and n = 10 PS2APP). (**b**) Bar histograms (mean ± SEM) and scatter plots reporting the discrimination index at 1 h and 24 h after learning in tORT in WT and PS2APP mice at 4, 6 and 8 months of age. 4mo and 6mo: n = 10 WT and 10 PS2APP mice; 8mo: n = 6 WT and n = 8 (1h) and 9 PS2APP (24h). (**c**) Same as (a) but for the classic ORT in WT (n = 6) and PS2APP (n = 6) mice at 6 and 8 months. (**d**) Box and scatter plots reporting spontaneous alternation rates in the Y-maze in WT and PS2APP mice at 6 and 8 months. Box plots report mean, median, 25th and 75th percentile, outliers. 6mo: n = 10 WT and 10 PS2APP mice; 8mo: n = 8 WT and n = 8 PS2APP. (**e**) Left, learning curves in the MWM for WT (n = 8) and PS2APP (n = 8) mice at 8 months (mean ± SEM). Right, time spent in the different quadrants during the probe trial (mean ± SEM). Full color column corresponds to the target quadrant. Individual values are reported as symbols connected by a dashed line. *$p < 0.05$, ***$p < 0.001$. Two-tailed Student t-test (a, b, c, d) and two-way RM ANOVA (e). Source data are provided as a Source Data file.

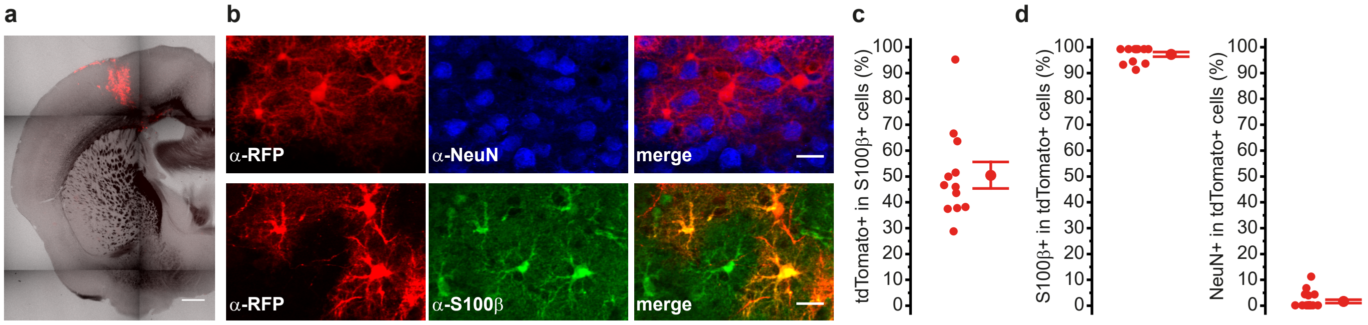

Supplementary Fig. 7. Astrocyte selective expression of mSTIM1 LV in the SSCx of 6mo PS2APP mice. (**a**) Intracortical mSTIM1 LV injection results in mSTIM1 LV expression in a restricted portion of the SSCx as shown in this image (representative of 3 mice). Scalebar 500 μm. (**b**) The images show that mSTIM1 LV expression is specific for astrocytes. tdTomato was stained with α-RFP, S100β (green) is a marker for astrocytes and NeuN (blue) is a marker for neurons. Scalebar 20 μm. (**c**) Scatter graph showing mSTIM LV penetrance to S100β+ astrocytes (mean ± SEM). n = 12 fields from 2 mice. (**d**) Left, scatter graph showing that 98% of mSTIM1 LV positive cells are also S100β+ (mean ± SEM). n = 12 fields from 2 mice. Right, scatter graph showing that only 1.5% of mSTIM1 LV positive cells are also NeuN+ (mean ± SEM). n = 20 fields from 2 mice. Source data are provided as a Source Data file.
