# Supplementary material for: Rescue of astrocyte activity by the calcium sensor STIM1 restores long-term synaptic plasticity in female mice modelling Alzheimer’s disease
Source: Nat Commun. 2023 Mar 22;14:1590. doi: 10.1038/s41467-023-37240-2 (PMC10033875; doi:10.1038/s41467-023-37240-2)
Supplement: Supplementary file 3 — Reporting Summary [file 41467_2023_37240_MOESM3_ESM.pdf]

nature portfolio

Corresponding author(s): Giorgio Carmignoto, Cristina Fasolato, Micaela Zonta

Last updated by author(s): Feb 17, 2023

Reporting Summary

Nature Portfolio wishes to improve the reproducibility of the work that we publish. This form provides structure for consistency and transparency in reporting. For further information on Nature Portfolio policies, see our Editorial Policies and the Editorial Policy Checklist.

Statistics

For all statistical analyses, confirm that the following items are present in the figure legend, table legend, main text, or Methods section.

n/a	Confirmed	
☐	☒	The exact sample size (*n*) for each experimental group/condition, given as a discrete number and unit of measurement
☐	☒	A statement on whether measurements were taken from distinct samples or whether the same sample was measured repeatedly
☐	☒	The statistical test(s) used AND whether they are one- or two-sided<br>*Only common tests should be described solely by name; describe more complex techniques in the Methods section.*
☐	☒	A description of all covariates tested
☐	☒	A description of any assumptions or corrections, such as tests of normality and adjustment for multiple comparisons
☐	☒	A full description of the statistical parameters including central tendency (e.g. means) or other basic estimates (e.g. regression coefficient) AND variation (e.g. standard deviation) or associated estimates of uncertainty (e.g. confidence intervals)
☐	☒	For null hypothesis testing, the test statistic (e.g. *F*, *t*, *r*) with confidence intervals, effect sizes, degrees of freedom and *P* value noted<br>*Give P values as exact values whenever suitable.*
☒	☐	For Bayesian analysis, information on the choice of priors and Markov chain Monte Carlo settings
☒	☐	For hierarchical and complex designs, identification of the appropriate level for tests and full reporting of outcomes
☐	☒	Estimates of effect sizes (e.g. Cohen's *d*, Pearson's *r*), indicating how they were calculated

Our web collection on statistics for biologists contains articles on many of the points above.

Software and code

Policy information about availability of computer code

Data collection	SciScan 1.2, Clampex 10.5, EthoVision XT14 software (Noldus), Leica Application Suite (LAS) software
Data analysis	Clampfit 11.1, Origin 2018, Microsoft Excel 2016, ImageJ 1.51n, EthoVision XT14 software (Noldus), GECIquant plugin, MatLAB 7.6.0 R2008 A, AstroResp

For manuscripts utilizing custom algorithms or software that are central to the research but not yet described in published literature, software must be made available to editors and reviewers. We strongly encourage code deposition in a community repository (e.g. GitHub). See the Nature Portfolio guidelines for submitting code & software for further information.

Data

Policy information about availability of data

All manuscripts must include a data availability statement. This statement should provide the following information, where applicable:

- Accession codes, unique identifiers, or web links for publicly available datasets
- A description of any restrictions on data availability
- For clinical datasets or third party data, please ensure that the statement adheres to our policy

The data that support the findings of this study are available from the corresponding author upon reasonable request.

Human research participants

Policy information about studies involving human research participants and Sex and Gender in Research.

Reporting on sex and gender	N/A
Population characteristics	N/A
Recruitment	N/A
Ethics oversight	N/A

Note that full information on the approval of the study protocol must also be provided in the manuscript.

Field-specific reporting

Please select the one below that is the best fit for your research. If you are not sure, read the appropriate sections before making your selection.

[x] Life sciences [ ] Behavioural & social sciences [ ] Ecological, evolutionary & environmental sciences

For a reference copy of the document with all sections, see nature.com/documents/nr-reporting-summary-flat.pdf

Life sciences study design

All studies must disclose on these points even when the disclosure is negative.

Sample size	No statistical methods were used to predetermine sample size. The sample size was chosen based on our previous experience and on our previous publications using similar technical approaches (Requie et al. 2022 Nature Neuroscience; Mariotti et al. 2018 Nature Communications; Sansevero et al. 2016 Experimental Neurology).
Data exclusions	Exploration thresholds were utilized for ORT and Tactile ORT: at least 8 seconds of total exploration during the learning phase and at least 2 seconds for each object during the test phases. Two animals did not reach the first threshold and therefore have been excluded.
Replication	To obtain reproducible findings all experiments were repeated, as indicated in text and/or figure legends.
Randomization	Allocation was random.
Blinding	In behavior experiments, data collection and analysis were blinded to investigators. In immunohistochemistry, calcium imaging and electrophysiology experiments, data collection was not blinded to investigators but data analysis was blinded to investigators.

Reporting for specific materials, systems and methods

We require information from authors about some types of materials, experimental systems and methods used in many studies. Here, indicate whether each material, system or method listed is relevant to your study. If you are not sure if a list item applies to your research, read the appropriate section before selecting a response.

Materials & experimental systems

n/a	Involved in the study	
[ ]	[x]	Antibodies
[x]	[ ]	Eukaryotic cell lines
[x]	[ ]	Palaeontology and archaeology
[ ]	[x]	Animals and other organisms
[x]	[ ]	Clinical data
[x]	[ ]	Dual use research of concern

Methods

n/a	Involved in the study	
[x]	[ ]	ChIP-seq
[x]	[ ]	Flow cytometry
[x]	[ ]	MRI-based neuroimaging

Antibodies

Antibodies used	Primary antibodies:<br>anti-S100β (RRID: AB_2620025, 1:300 polyclonal in guinea pig, Synaptic System, Germany, 287004, Lot n. 1-9); anti-GFAP (RRID: AB_10013382, 1:300 polyclonal in rabbit, Dako Agilent, Denmark, Z0334, Lot n. 00005193); anti β-Amyloid 17-24 (RRID: AB_2734548, 1:500 monoclonal in mouse, 4G8 clone, Biolegend, SanDiego CA, 800712, Lot n. B286227); anti-STIM1 (RRID: AB10828699, 1:400 monoclonal in rabbit, clone D88E10, Cell Signaling, 5668); anti-NeuN (RRID: AB_2298772, 1:100 monoclonal in mouse, A60 clone,

	Merck-Millipore MAB377, Lot n. 3061189); anti-RFP (RRID: AB_2209751, 1:1000 in rabbit, Rockland, 600-401-379, Lot n. 42872).<br>Secondary antibodies:<br>Alexa Fluor-488 conjugated (donkey anti-mouse, RRID: AB_141607, 1:500, A21202, Invitrogen Thermo Fisher Scientific, Lot n. 1741782; donkey anti-rabbit, RRID: AB_2535792, 1:500, A21206, Invitrogen Thermo Fisher Scientific, Lot n. 2289872; goat anti-guinea pig, RRID: AB_2534117, 1:500, A11073, Lot n. 982288, Invitrogen Thermo Fisher Scientific), Alexa Fluor-556 conjugated (donkey anti-mouse, RRID: AB_2534012, 1:500, A10036, Invitrogen Thermo Fisher Scientific, Lot n. 2160040), Alexa Fluor-555 conjugated (donkey anti-rabbit, RRID: AB_162543, 1:500, A31572, Invitrogen Thermo Fisher Scientific, Lot n. 2286312), Alexa Fluor-633 conjugated (F (ab')2-Goat anti-Mouse, RRID: AB_2535720, 1:500, A21053, Invitrogen Thermo Fisher Scientific, Lot n. 431664).<br>Nuclei were stained with To-Pro-3 (1:1000, T3605, InvitrogenThermo Fisher Scientific, Lot n. 1212122).
Validation	For immunohistochemical stainings commercial, broadly used antibodies have been employed.<br>anti-S100β: 17 references reported on manufacturer's website (https://sysy.com/product/287004).<br>anti-GFAP: excellent specificity and optimization for IHC reported on manufacturer's website (https://www.agilent.com/en/product/immunohistochemistry/antibodies-controls/primary-antibodies/glial-fibrillary-acidic-protein-(concentrate)-76683#features).<br>anti β-Amyloid 17-24: 99 publications reported on manufacturer's website (https://www.biolegend.com/de-at/search-results/purified-anti-beta-amyloid-17-24-antibody-11233).<br>anti-STIM1: 74 citations reported on manufacturer's website and the specificity section reports that the antibody does not cross-react with STIM2 (https://www.cellsignal.com/products/primary-antibodies/stim1-d88e10-rabbit-mab/5668).<br>anti-NeuN: more than 100 references reported on manufacturer's website (https://www.merckmillipore.com/IT/it/product/Anti-NeuN-Antibody-clone-A60,MM_NF-MAB377#anchor_REF).<br>anti-RFP: 927 references reported on manufacturer's website (https://www.rockland.com/categories/primary-antibodies/rfp-antibody-pre-adsorbed-600-401-379/).

Animals and other research organisms

Policy information about studies involving animals; ARRIVE guidelines recommended for reporting animal research, and Sex and Gender in Research

Laboratory animals	C57BL/6J, PS2APP (B6.152H), PS2.30H (PS2-N141I), APPSwe female mice at 3, 4, 6 and 8 months of age were used. For transgenic mice it has been indicated the reference where these mice have been described. Mice were housed under a 12h light-dark cycle (7am to 7pm light), with a room temperature of 22°C and humidity of 60%.
Wild animals	N/A
Reporting on sex	Our findings apply to only female mice. In the M&M section we explained the reason behind this choice.
Field-collected samples	N/A
Ethics oversight	All experimental procedures were performed according to the European 331 Committee guidelines (decree 2010/63/CEE) and the Animal Welfare Act (7 USC 2131), in compliance with the ARRIVE guidelines, and were approved by the Animal Care Committee of the University of Padua and the Italian Ministry of Health (authorization decrees 461/2017-PR, 929/2016-PR and 789/2019-PR).

Note that full information on the approval of the study protocol must also be provided in the manuscript.
